# Photoperiodic Changes in Both Hypothalamus Neurotransmitters and Circulating Gonadal Steroids Metabolomic Profiles in Relation to Seasonal Reproduction in Male Quail

**DOI:** 10.3389/fphys.2022.824228

**Published:** 2022-03-25

**Authors:** Yanglong Xu, Danli Jiang, Jiaxin Liu, Yuting Fu, Yan Song, Di Fan, Xuefei Huang, Sui Liufu, Jianqiu Pan, Hongjia Ouyang, Yunbo Tian, Xu Shen, Yunmao Huang

**Affiliations:** ^1^College of Animal Science and Technology, Zhongkai University of Agriculture and Engineering, Guangzhou, China; ^2^Guangdong Province Key Laboratory of Waterfowl Healthy Breeding, Guangzhou, China

**Keywords:** photoperiod, neurotransmitters, steroid biosynthesis, gene expression, quail

## Abstract

Both hypothalamic neurotransmitters and serum steroid hormones are impacted by photoperiod and have effects on physiology and seasonal reproductive. However, the relationship between circulating gonadal steroids and hypothalamic neurotransmitters underlying different photoperiod is still unclear. To further understand the crosstalk of neurotransmitters and steroids in seasonal reproduction, metabolic changes of 27 neurotransmitters concentrated in hypothalamus tissues and 42 steroids hormones in serum were assessed during two artificial photoperiodic programs. The results showed that photoperiod induce testicular atrophy and recrudescence. In L-to-S groups, significantly decreased levels of testosterone concentration were found in serum (*P* < 0.001) and increased 11-Dehydrocorticosterone (*P* < 0.05); Testosterone were almost undetectable at SD_14d. In addition, the hypothalamus exhibited significantly increased arginine and 4-aminobutyric acid (GABA) concentration and decreased serotonin and epinephrine content (*P* < 0.01 or *P* < 0.05). Accordingly, serum testosterone and androstenedione became detectable at LD_3d in the S-to-L group and were markedly increase at LD_7d. Furthermore, Serum androstenedione showed a significant increase with long light expose (*P* < 0.01). Additionally, the hypothalamus exhibited both significantly increased L.Tryptophan and phenylalanine concentration, as well as decreased L-glutamine and L-glutamine.acid content (*P* < 0.01 or *P* < 0.05). Serotonin metabolism showed significant differences between L-to-S group and S-to-L group. Furthermore, in the correlation analysis, serum testosterone had a positive correlation with 5-Hydroxyindole-3-acetic acid (5-HIAA), while Androstenedione was significantly negative with L.Tryptophan in L-to-S (*P* < 0.05). However, in S-to-L group, serum testosterone showed strong negative correlation with both serotonin and 5-HIAA (*P* < 0.05), but positive correlation with L.Tryptophan (*P* < 0.01), while Androstenedione was significantly negative correlation with both serotonin (*P* < 0.05) and L-Glutamine (*P* < 0.01). Photoperiod also had significant effects on the mRNA expression. We found significant differences in gene expression patterns of both serotonin signaling and steroid biosynthesis, while *MAOB*, *NR5A1*, and *3*β*-HSD* showed an opposite tendency between two groups. Taken together, our results revealed that circulating gonadal steroids and hypothalamic neurotransmitters were significantly impact quail’s seasonal reproduction. Circulating gonadal steroids have different effects on neurotransmitter at different photoperiodism, which may coordinately influence the seasonal reproduction of quails.

## Introduction

Seasonal breeding is a reproductive strategy for wild animals that is day-length (photoperiod) dependent (Bünning, 1960). The molecular basis for the regulation of seasonal reproduction is still unknown. However, it is thought to be primarily regulated by the hypothalamus-pituitary-gonadal (HPG) axis in birds (Shinomiya et al., 2014; Nishiwaki-Ohkawa and Yoshimura, 2016). In quail, deep brain photoreceptors receive and transmit light signals to the pars tuberalis, which induces the secretion of thyroid-stimulating hormone (Halford et al., 2009; Nakane et al., 2010; Nakane and Yoshimura, 2011). Thyroid-stimulating hormone activates the type 2 deiodinase (dio2)/type 3 deiodinase (dio3) switching system and induces local thyroid hormone release in the mediobasal hypothalamus (Ono et al., 2008). Thyroid hormone then induces morphological changes in the terminals of gonadotropin-releasing hormone (GnRH) neurons and facilitates gonadotropin secretion from the pituitary gland (Yamamura et al., 2004; Shinomiya et al., 2014; Nishiwaki-Ohkawa and Yoshimura, 2016). Nevertheless, how photoperiodism initiates the complex neuroendocrine signaling cascade that ultimately leads to an activated reproductive axis is still a hot topic for researchers of seasonal reproduction in mammals and birds.

Seasonal reproductive activity is driven by photostimulation and depends on neuroendocrine regulation, with striking changes of hormones in the hypothalamus-pituitary-gonadal (HPG) axis to initiate and maintain gonad development (Mauro et al., 1989; El Halawani and Rozenboim, 1993; Dunn et al., 2004; Johnson, 2015). Thyroid hormones are considered to be critical signaling molecules regulating avian reproductive seasonality (Nicholls et al., 1988; Yoshimura et al., 2003; Nakao et al., 2008). In birds, activity of the HPG axis is strictly controlled by the levels of gonadotropin releasing hormone (GnRH). GnRH neurons in the mediobasal hypothalamus (MBH) have dynamic morphological plasticity in response to changes in photoperiod, which could modulate seasonal GnRH secretion from the hypothalamus (Jansen et al., 2003; Yamamura et al., 2004; Lehman et al., 2010). Thyroid hormone changes markedly from the reproductively active phase to the inactive phase in birds (Donham, 1979), and T3 controls the seasonal pulse release of GnRH (Hanon et al., 2008; Nakane and Yoshimura, 2010). Seasonal reproductive activity in the HPG axis is striking with GnRH/GnIH release and gonadal steroid negative feedback (Kriegsfeld et al., 2015). Changes in plasma concentration of prolactin (PRL) and luteinizing hormone (LH) are also reported to be related to seasonal reproduction in the Magang goose (Huang et al., 2008).

Steroid modulation of neurotransmitter function to alter reproductive behavior in rodents (McCarthy and Pfaus, 1996). Neurotransmitters serve as key components of neuroendocrine circuitry and have been found to play important roles in circadian rhythm, reproduction, and adaptation to environmental changes. On another hand, the endocrine system can be modulated by neurotransmitters (Kang et al., 2009). Serotonin (5-HT) is a mono-amine inhibitory neurotransmitter, that plays an important role in avian reproduction. Serotonin’s effect on reproductive tissues plays a prominent role in gonadotropin secretion (Prasad et al., 2015), prolactin release (El Halawani et al., 1995; Bakken et al., 2014), and steroidogenesis (Niaraki et al., 1982; Frungieri et al., 1999). Serotonin is associated with light exposure (Nakayama et al., 2020) and the effect of photoperiod and temperature can alter the production of serotonin. The serotonin system may also play a unique role in the control of seasonal reproduction (Abbas et al., 2011; Nakayama et al., 2020).

However, the intra-hypothalamic mechanisms underlying the actions of neurotransmitters on the reproductive axis remain unclear. The neurotransmitters influence testis function and, in turn, they are affected by testicular function through gonadal steroid secretion (Rasri et al., 2008). However, the crosstalk of neurotransmitters and steroids in seasonal reproduction under different photoperiod has remained not clear.

The quail is an ideal model for studying seasonal biology. Herein, we used male quail to investigate the impact of photoperiod on brain neuron transmitters and serum steroid hormones, as well the relationship between circulating gonadal steroids and hypothalamic neurotransmitters in quails underlying different photoperiod.

## Materials and Methods

### Animals and Light Schedules

**FIGURE 1:**
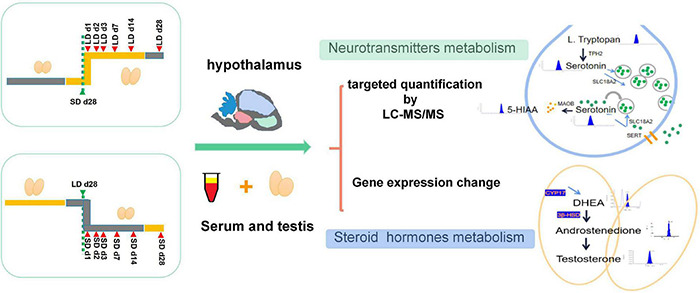
Experimental design of this study. All quails were randomly divided into two types of group, In S-to-L groups: samples were collected at SD_28d, LD_d1, LD_d2, LD_d3, LD_d7, LD_d14, and LD_d28; In the L-to-S groups, samples were collected at LD_d28, SD_d1, SD_d2, SD_d3, SD_d7, SD_d14, and SD_d28. Both hypothalamus homogenization and serum samples in the L-to-S and S-to-L groups were collected for metabolome analysis. Both hypothalamus tissue and testis in the L-to-S and S-to-L groups were collected for qPCR analysis.

Two hundred and forty 7-week-old male quails were pre-fed for 4 weeks with unlimited standard food and water at a constant room temperature of 21°C and light intensity of 245 lux under a 16 h light/8 h dark cycle. At 11 weeks of age, all quails were randomly divided into were divided into two types of group, with six birds in each group. The experimental schedules are shown in Figure 1. In the short-to-long (S-to-L) groups: quail were maintain under short daylength (SD) conditions [6 light (L):18 dark (D)] for 4 weeks and then transferred to long daylength (LD) conditions (20L:4D) for 4 weeks to stimulate testis recovery. Samples were collected at SD_28d, LD_d1, LD_d2, LD_d3, LD_d7, LD_d14, and LD_d28; In the L-to-S groups, quail were kept under LD conditions (20L:4D) for 4 weeks and then transferred to SD conditions (6L:18D) for 4 weeks to induce testicular atrophy. Samples were collected at LD_d28, SD_d1, SD_d2, SD_d3, SD_d7, SD_d14, and SD_d28. All samples were collected at 8 h after lights-on zeitgeber time 8 (ZT8), which for SD birds was 2 h after dark onset, and for LD birds 12 h before dark onset (lights-on was the same for both LD groups and SD groups). All experimental protocols were approved by the Animal Experiment Committee of Zhongkai University of Agriculture and Engineering (No. 2020090909). Testicular Morphology and histological changes, steroid metabolism, neurotransmitter concentration, and key gene expression levels were investigated.

### Target Metabolomics for Both Neurotransmitters and Steroids

Blood samples were collected and serum was produced by centrifugation at 4,000 rpm at 4°C for 20 min. The serum samples were used to quantify 42 steroids via UPLC-HRMS analysis (Supplementary Table 1). Each frozen hypothalamus sample was immediately frozen in liquid nitrogen until homogenization. Hypothalamus neurotransmitter metabolites were measured. Standard solutions of neurotransmitters (*n* = 27) with different concentration gradients were prepared (Supplementary Table 2).

Crushed hypothalamic tissues (30 mg) in 10 μL ascorbic acid (Concentration 10 mg/mL) (Merck, KGaA, Germany), add 400 μL methanol solution and vortex for 1 min; The hypothalamic homogenate was placed in an ice bath for ultrasonic crushing for 32 s; Supernatant were harvested by centrifugation at 13,000 r/min for 5 min at 4°C, and transfered to a clear tube. Add 100 μL methanol to the residue and vortex for 1 min, centrifuge again, harvested the supernatant, and combined the twice supernatant, dried with nitrogen. Redissolved by add 500 μL methanol solution at 20%, centrifuge at 13,000 r/min for 5 min at 4°C to extract and enrich the hypothalamic neurotransmitter, supernatants were transfered for machine analysis.

The frozen serum was removed from the ultra-low temperature refrigerator, vortexed and mixed for 10 s after thawing, and 100 μL of serum was added to 400 μL of methanol. Shaking for 5 min and standing for 5 min was repeated twice. The mixture was then centrifuged for 10 min at 12,000 r/min and 4°C, and the supernatant was collected. The supernatant was reconstituted by adding 100 μL of methanol solution and then centrifuged at 12,000 r/min for 1 min at 4°C. Aliquots of 80 μL of supernatant and use AB SCIEX QTRAP 6500 + detection platform to determine.

AB SCIEX QTRAP LC-MS/MS detection platform was used to detect the metabolites in the sample and quantify the content of both steroids in serum and the neurotransmitter in hypothalamic tissues. Standard solutions were prepared in the following volumes: 0.01, 0.02, 0.05, 0.1, 0.2, 0.5, 1, 2, 5, 10, 20, 50, 100, 200, 500, and 1,000 ng/mL. The data were processed by Analyst Software 1.6, ACD/Chem Sketch, and Excel 2010 software. For detected steroids, the Kinetex C18 column (1.7 μm, 100 mm × 2.1 mm i.d.) was used at a controlled temperature of 40°C; phase A consisted of acetonitrile/water and phase B was acetonitrile/isopropyl alcohol. A flow rate of 0.35 mL/min was used with an injection volume of 5 μL. For detected neurotransmitter, the Kinetex C18 100A column (2.6 μm, 50 mm × 2.1 mm i.d.) was used at a controlled temperature of 30°C; Phase A consisted of 0.05% formic acid, and phase B was 0.05% formic acid/acetonitrile. A flow rate of 0.4 mL/min was used with an injection volume of 10 μL.

Under mass spectrometry conditions, For detected steroids, the ion mode was ESI-/ESI +, using the following parameters: ion source temperature = 500°C; ion spray voltage = −4,500/ + 5,500 V; curtain gas (nitrogen) = 35 psi; atomizing gas (GS 1) = 50 psi; auxiliary gas (GS 2) = 60 psi. For detected neurotransmitter, the scan type was multiple reaction monitoring (MRM), using the following parameters: ion source temperature = 300°C; atomizing gas (GS 1) = 60 psi; auxiliary gas (GS 2) = 75 psi. All data were collected using Sciex Analyst 1.6 software performed at Wuhan MetWare Biotechnology Co., Ltd.

### Gene Expression Analysis

Total RNA was extracted from the hypothalamus tissues using a TRIzol reagent kit (Invitrogen Inc., Minneapolis, MN, United States) and reverse-transcribed into cDNA. qRT-PCR reactions were performed in AB7500 using PowerUP SYBR Green Master MIX (Thermo Fisher Scientific, Waltham, UAB). Primers were designed using Primer 3.0 software and then synthesized by Sangon Biotech (Shanghai) Co., Ltd. (Supplementary Table 3). All samples were amplified for 40 cycles and the relative expression level of both the serotonin metabolism pathway (*TPH2*, *SERT*, *MaoB*, and *SCL18A2*) and the steroid metabolism pathway (*NR5A1*, *3*β*-HSD*, *CYP11A1*, *CYP17A1*, and *StAR*) were calculated using the ^ΔΔ^C_t_ -method.

### Data Analysis

For Gene expression data: Statistical significance was determined by ANOVA with Tukey’s multiple comparisons using Prism 7 (GraphPad Software Inc., La Jolla, CA, United States), *P* < 0.05 were considiered as statistically significant. For targeted metabolomics data, Graphics for both steroid concentration and neurotransmitter concentration were plotted using the ggplot2 package, ANOVA analysis was performed to estimate whether steroids (or neurotransmitters concentration) had a significant time-point dependent effect; Correlation was assessed by Pearson’s test given in the R program, *P* < 0.05 were considiered as statistically significant.

## Results

### Influence of Photoperiodism on Testicular Function

**FIGURE 2:**
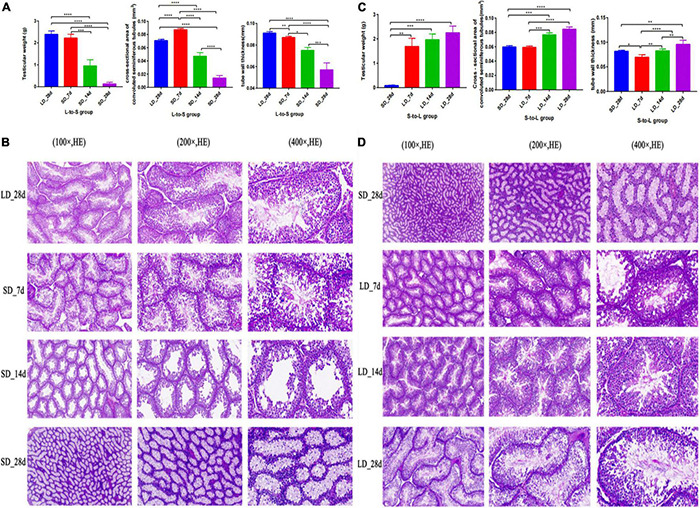
Testis phenotype change and histology of the testes in response to photoperiodism in quail. Statistical significance was determined by ANOVA with Tukey’s multiple comparisons*** means *P* < 0.001, ** means *P* < 0.01, * means *P* < 0.05. In L-to-S group, the testicular weights, cross-section area, and wall thickness were markedly decreased at the end of short days, and achieve the minimum in SD_28d **(A)**. In L-to-S group, histology of testis showed degenerative of germ cells and Leydig cells **(B)**. In S-to-L group, the highest testicular weights, cross-section area, and wall thickness were observed at the end of long days, and reached a maximum in LD_28d **(C)**. In contrast, in S-to-L group, histological structure of quail’s testis showed the seminiferous tubule consists of sperm, spermatogenic cells and sertoli cell, as well as leydig cells are found adjacent to the seminiferous tubules in the testicle **(D)**. ****p < 0.0001.

Testicular function was changed by light duration (Figure 2). We observed a marked decrease in testicular weight, cross-section area, and wall thickness when the light schedule changed from LD to SD; both tubule diameter and epithelial height displayed a drastic decline (*P* < 0.05, Figure 2A). Quail showed maximal testicular development after a long light regime (20L:4D), compared with the short-day regime. A notable increase in testicular weight, cross-section area, and wall thickness were observed when the light schedule changed from SD to LD; both the diameter and the epithelial height of the tubules were also observed to increase significantly (*P* < 0.05, Figure 2B). Therefore, short days induced testis regression while long days stimulated testes development.

Histological analysis of the test is showed that light exposure could directly influence spermatogenesis in quail. In L-to-S group, histology of testis showed degenerative of germ cells and Leydig cells (Figure 2C). In contrast, histological structure of quail’s testis showed development of spermatogonia, the seminiferous tubule consists of sperm, spermatogenic cells and sertoli cell, as well as leydig cells are found adjacent to the seminiferous tubules in the testicle during prolonged light in S-to-L group (Figure 2D).

### Photoperiod Effect on Serum Steroid Hormone Metabolism and Gene Expression Level on Steroid Metabolism Pathways

**FIGURE 3:**
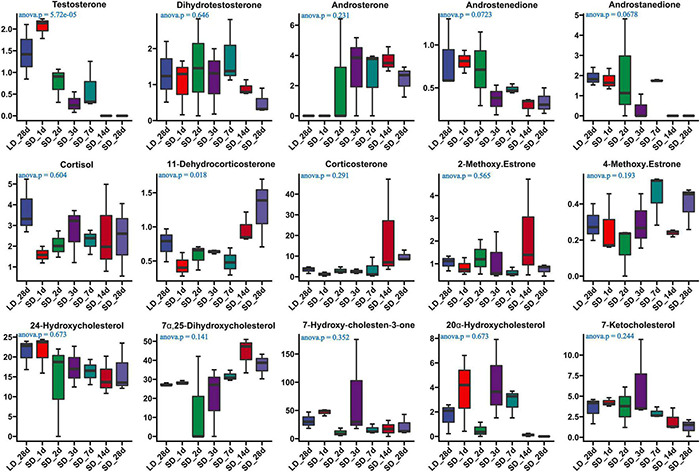
Content of serum steroids and their related metabolites in quail of the long-to-short (L-to-S) group. ANOVA analysis was performed to estimate whether steroids concentration had a significant time-point dependent effect in L-to-S group, *P* < 0.05 was considered statistically significant. Both 11- dehydrocorticosterone and testosterone show significant changes.

**FIGURE 4:**
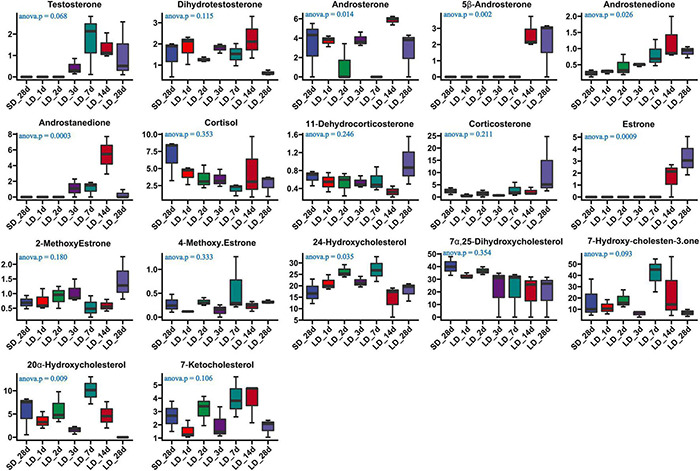
Content of serum steroids and their related metabolites in quail of the short-to-long (S-to-L group). ANOVA analysis was performed to estimate whether steroids concentration had a significant time-point dependent effect in S-to-L group. *P* < 0.05 was considered statistically significant. Androstenedione show significant changes.

The effect of photoperiod on steroid metabolism in the testis was studied (Supplementary Tables 4, 5). There was a significantly reduce concentration of testosterone in serum in response to SD conditions as compared to LD (*P* < 0.05). Both androstenedione and androstanedione showed a gradual decline under chronic short-day conditions, but this was not significant (Figure 3). The quail with abnormal steroid metabolism had almost undetectable concentrations of testosterone and androstanedione indicating the loss of reproductive function. Male quail quickly lost their testicular function when transferred to SD conditions for 14 d. In contrast, LD induces a rapid increase of both testosterone and androstenedione concentrations compared to SD, this difference was significant in androstenedione. The increasing tendency of both testosterone and androstenedione was indicated that both testosterone and androstenedione production was maximal at LD_7d. On the other hand, we found quail regained their testicular function at LD_d3 despite and androstenedione production being induced by long light exposure at LD_d1 (Figure 4).

**FIGURE 5:**
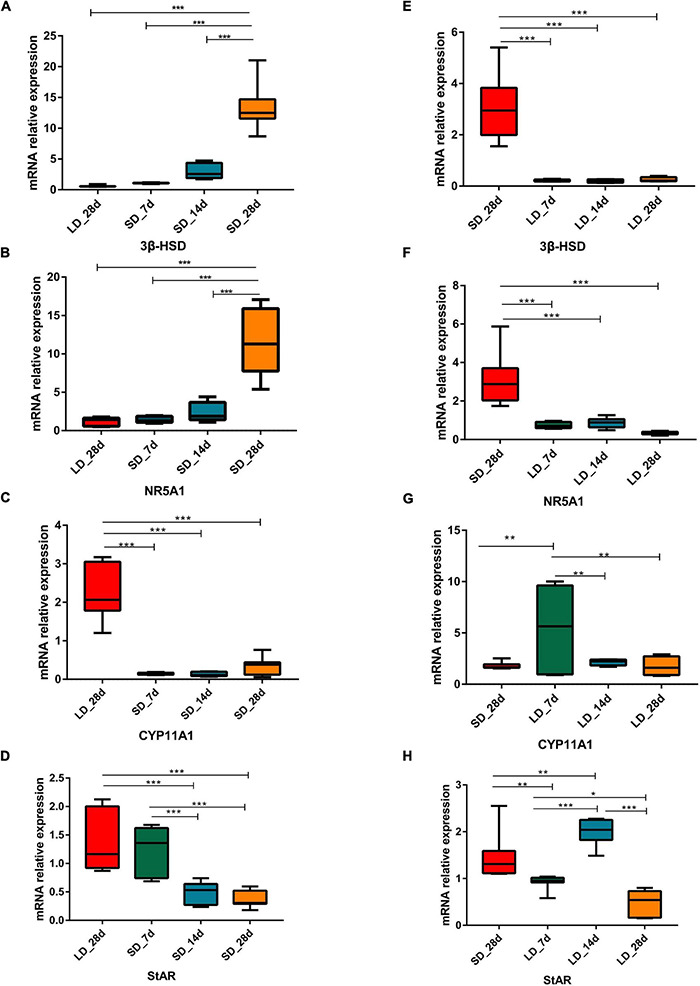
Photoperiodism regulated gene expression level in steroid biosynthesis pathway. **(A–D)** Real-time PCR quantification of expression of genes involved in steroid biosynthesis pathway in testis in L-to-S group: 3ß-HSD **(A)**, NR5A1 **(B)**, CYP11A1 **(C)**, and StAR **(D)**. **(E–H)** Real-time PCR quantification of expression of genes involved in steroid biosynthesis pathway in testis in S-to-L group: 3ß-HSD **(E)**, NR5A1 **(F)**, CYP11A1 **(G)**, and StAR **(H)**. Data are presented as 2^−ΔΔCT^ method (normalized to house keeping gene GAPDH). Statistical significance was determined by ANOVA with Tukey’s multiple comparisons, *** means *P* < 0.001, ** means *P* < 0.01, * means *P* < 0.05.

Gene expression levels in steroid metabolism pathways were influenced by photoperiod. Both *NR5A1* and *3*β*-HSD* showed a consistent pattern of decline during long light exposure. Both *CYP11A1* and *CYP17A1* were strongly increased at LD_7d (*P* < 0.01 or *P* < 0.05). However, gene expression levels were intensified by decreased day length. Both *NR5A1* and *3*β*-HSD* showed consistently up-regulated patterns when day length was artificially shortened. The expression level of *CYP11A1*, *CYP17A1*, and *StAR* were suppressed by short days (Figure 5).

### Photoperiod Effect on Hypothalamus Neurotransmitters and Gene Expression Level on the Serotonin Metabolism Pathway

**FIGURE 6:**
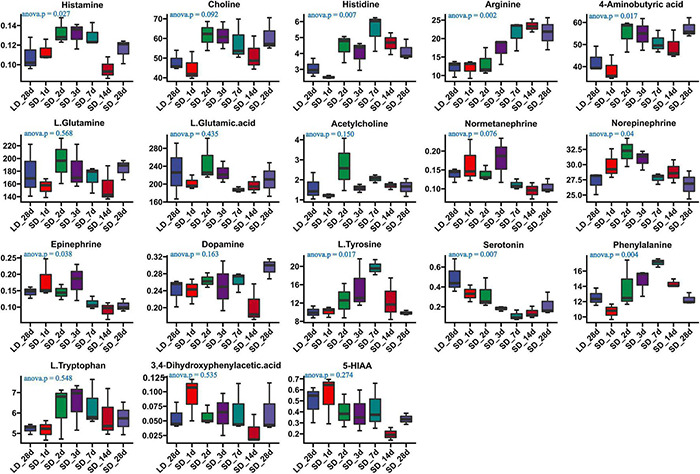
Content of neurotransmitters and their related metabolites in quail hypothalamus of the long-to-short (L-to-S) group. ANOVA analysis was performed to estimate whether neurotransmitters concentration had a significant time-point dependent effect in L-to-S group. *P* < 0.05 was considered statistically significant. Histamine, arginine,4-aminobutyric acid (GABA), norepinephrine, phenylalanine, epinephrine, and serotonin show significant changes between long daylight (LD) to short daylight (SD).

We investigated the effects of photoperiod on hypothalamus neurotransmitter content when the photoperiod change from LD to SD (Supplementary Table 6). Our results indicated that the content of histamine, histidine, arginine, GABA, Norepinephrine, L.Tyrosine, Epinephrine, phenylalanine and serotonin were significantly changed in the hypothalamus when quail were transformed from LD to SD (Figure 6). An progressively increase of arginine was observed in SD, and more notably, a progressively decrease of serotonin was observed in SD, indicating that SD promoted the metabolism of serotonin. However, there were no significantly changed of both tryptophan and 5-HIAA. Short day exposure resulted in dynamic changes of serotonin in the hypothalamus.

**FIGURE 7:**
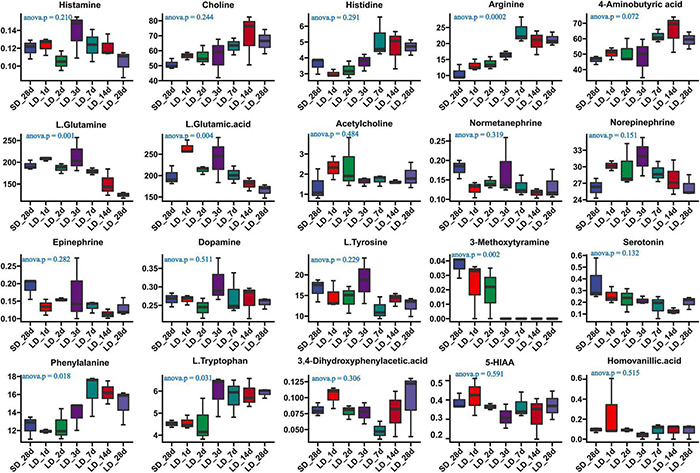
Content of neuron-transmitters and their related metabolites in quail hypothalamus of the short-to-long (S-to-L) group. ANOVA analysis was performed to estimate whether neurotransmitters concentration had a significant time-point dependent effect in S-to-L group. *P* < 0.05 was considered statistically significant. Arginine, 4-aminobutyric acid (GABA), phenylalanine, L-glutamine, L-glutamine.acid and L.Tryptophan show significant changes from short daylight (SD) to long daylight (LD).

We also investigated the long-day effects of photoperiodism on hypothalamus neurotransmitter content when the photoperiod changed from SD to LD (Supplementary Table 7). The comparison results from the measurement of the neurotransmitters associated with the mechanism of reproductive inactivity are shown in Figure 7. Our results indicated that the content of arginine, L-glutamine, L-glutamine acid, phenylalanine and L.Tryptophan were significantly changed in the hypothalamus when quail were transformed from SD to LD. A progressively increase in both phenylalanine and L.Tryptophan was observed in LD; more notably, a sharp decrease in L-glutamine was observed in SD, indicating that LD promoted the metabolism of L-glutamine, and induce the increase of L.Tryptophan (Figure 7). However, there were no significant changes in serotonin and 5-HIAA metabolism.

**FIGURE 8:**
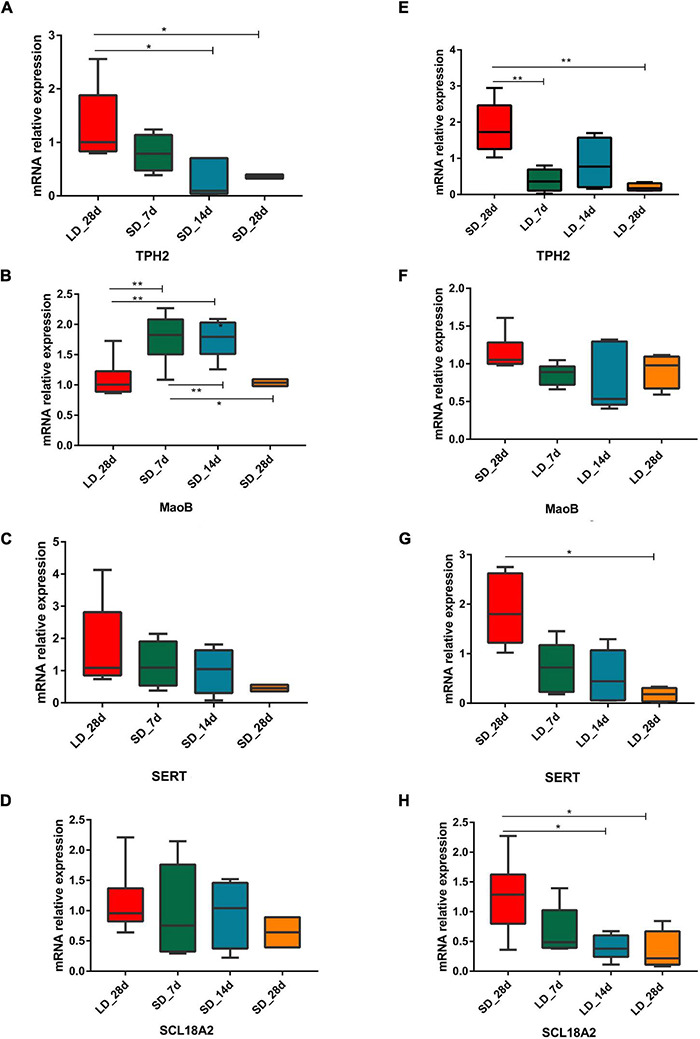
Photoperiodism regulated gene expression level in neurotransmitter metabolism pathway. **(A–D)** Real-time PCR quantification of expression of genes involved in serotonin metabolic pathway in hypothalamus in L-to-S group:TPH2 **(A)**, MaoB **(B)**, SERT **(C)**, and SCL18A2 **(D)**. **(E–H)** Real-time PCR quantification of expression of genes involved in serotonin metabolic pathway in hypothalamus in S-to-L group: TPH2 **(E)**, MaoB **(F)**, SERT **(G)**, and SCL18A2 **(H)**. Data are presented as 2^−*Delta*ΔCT^ method (normalized to house keeping gene GAPDH). Statistical significance was determined by ANOVA with Tukey’s multiple comparisons, *** means *P* < 0.001, ** means *P* < 0.01, * means *P* < 0.05.

As a difference in serotonin metabolism was observed in our data, we investigated the role of short photoperiod on the expression of key genes in the hypothalamus after the transition of light conditions. We observed a significant decrease in mRNA expression levels of serotonin metabolism related genes in the hypothalamus. We also found that short days reduced the expression of *TPH2*, TPH2 gene showing differences in expression between long day and short days. Low expression levels were observed under chronic SD and showed a significant decrease when quail moved from LD to SD. However, we found that the expression of *MAOB* was LD-induced and it progressively increased at SD_7 and SD_14d (*P* < 0.01), compared with the expression of LD_28d (Figures 8A–D).

During transition from SD to LD, changes in gene expression took place. For each time point, excepting *MAOB*, the three serotonin metabolism related genes (*TPH2*, *SERT*, and *SLC18A2*) were considered as LD reducing genes, which showed a difference in expression between SD and LD; low expression levels were observed under chronic LD conditions and they were significantly decreased from when quail moved from LD to SD (Figures 8E–H).

### Correlation Between Neurotransmitter Metabolism and Serum Steroid Hormones

In the correlation analysis, In L-to-S group, serum testosterone showed a positive correlation with 5-HIAA (*r* = 0.44, *P* = 0.048), Epinephrine (*r* = 0.45, *P* = 0.041), and a negative correlation with GABA (*r* = −0.55, *P* = 0.010), Histidine (*r* = −0.51, *P* = 0.017) and Arginine (*r* = −0.60, *P* = 0.003); moreover, Androstenedione had a negative correlation with L.Tryptophan (*r* = −0.45, *P* = 0.040), Arginine (*r* = −0.58, *P* = 0.006); In S-to-L group, testosterone showed a negative correlation with progesterone 5-HIAA (*r* = −0.49, *P* = 0.025), serotonin (*r* = −0.52, *P* = 0.017), and was positively associated with GABA (*r* = 0.64, *P* = 0.002), Phenylalanine (*r* = 0.6, *P* = 0.004), L.Tryptophan (*r* = 0.59, *P* = 0.005), Histidine (*r* = 0.69, *P* = 0.0006), Choline (*r* = 0.60, *P* = 0.004)and Arginine (*r* = 0.68, *P* = 0.0007);Whereas Androstenedione had a positive correlation with Phenylalanine (*r* = 0.55, *P* = 0.010), Arginine (*r* = 0.50, *P* = 0.020) and showed negative correlation with Serotonin (*r* = −0.45, *P* = 0.041), L.Glutamine (*r* = −0.63, *P* = 0.002), L.Glutamic.acid (*r* = −0.47, *P* = 0.03) (Tables 1, 2).

**TABLE 1:** Correlation between neurotransmitter metabolism and serum steroid hormones in quail in L-to-S group.

Neurotransmitter	Steroid	L-to-S group	
		*r*	*p* value
5-Hydroxyindole-3-acetic acid	20-Hydroxycholesterol	0.55	0.0092**
5-Hydroxyindole-3-acetic acid	Testosterone	0.44	0.0481*
5-Hydroxyindole-3-acetic acid	7,25-Dihydroxycholesterol	−0.51	0.0194*
4-Aminobutyric.acid	Testosterone	−0.55	0.0105*
3,4-Dihydroxyphenyl acetic.acid	20-Hydroxycholesterol	0.6	0.0040**
Serotonin	Androsterone	−0.46	0.0352*
Phenylalanine	Androsterone	0.54	0.0115*
Phenylalanine	24-Hydroxycholesterol	−0.43	0.0499*
Normetanephrine	20-Hydroxycholesterol	0.78	<0.0001***
Normetanephrine	7-Ketocholesterol	0.68	0.0008***
Normetanephrine	7-Hydroxy.cholesten.3.one	0.61	0.0034**
Norepinephrine	4-Methoxy.Estrone	−0.46	0.0376*
Norepinephrine	7,25-Dihydroxycholesterol	−0.49	0.0226*
L.Tyrosine	Androsterone	0.47	0.0300*
L.Tryptophan	Androstenedione	−0.45	0.0401*
L.Tryptophan	24-Hydroxycholesterol	−0.46	0.0380*
Histidine	Androstenedione	−0.46	0.0364*
Histidine	Testosterone	−0.51	0.0175*
Histamine	Corticosterone	−0.45	0.0416*
Histamine	7,25-Dihydroxycholesterol	−0.54	0.0124*
Epinephrine	20-Hydroxycholesterol	0.77	<0.0001***
Epinephrine	7-Ketocholesterol	0.65	0.0014**
Epinephrine	7-Hydroxy.cholesten.3.one	0.56	0.0090**
Epinephrine	Testosterone	0.45	0.0402*
Arginine	Androsterone	0.48	0.0291*
Arginine	Androstanedione	−0.57	0.0074**
Arginine	Androstenedione	−0.58	0.0057**
Arginine	Testosterone	−0.6	0.0039**
Acetylcholine	7,25-Dihydroxycholesterol	−0.54	0.0123*

Correlations were analyzed using Pearson’s test. Pearson’s r means Pearson correlation coefficient. * means P < 0.05, ** means P < 0.01, *** means P < 0.001.

**TABLE 2:** Correlation between neurotransmitter metabolism and serum steroid hormones in quail in S-to-L group.

Neurotransmitter	Steroid	S-to-L group	
		*r*	*p* value
5-Hydroxyindole-3-acetic acid	Testosterone	−0.49	0.0246*
4-Aminobutyric.acid	Testosterone	0.64	0.0017**
3-Methoxytyramine	Testosterone	−0.49	0.0245*
3-Methoxytyramine	7α,25-Dihydroxycholesterol	0.5	0.0204*
3-Methoxytyramine	Androstenedione	−0.6	0.0038**
3,4-Dihydroxyphenylacetic acid	Corticosterone	0.45	0.0405*
3,4-Dihydroxyphenylacetic acid	7-Ketocholesterol	−0.5	0.0208*
3,4-Dihydroxyphenylacetic acid	11-Dehydrocorticosterone	0.56	0.0079**
Serotonin	Androstenedione	−0.45	0.0410*
Serotonin	Testosterone	−0.52	0.0168*
Phenylalanine	Androstanedione	0.48	0.0293*
Phenylalanine	5β-Androsterone	0.49	0.0239*
Phenylalanine	Androstenedione	0.55	0.0101*
Phenylalanine	Testosterone	0.6	0.0042**
L.Tryptophan	Testosterone	0.59	0.0045**
L.Glutamine	Corticosterone	−0.52	0.0159*
L.Glutamine	Androstenedione	−0.63	0.0022**
L.Glutamine	Estrone	−0.65	0.0016**
L.Glutamine	5β-Androsterone	−0.7	0.0005***
L.Glutamic.acid	Estrone	−0.44	0.0435*
L.Glutamic.acid	Androstenedione	−0.47	0.0296*
L.Glutamic.acid	Corticosterone	−0.51	0.0173*
L.Glutamic.acid	5β-Androsterone	−0.52	0.0161*
Histidine	7-Hydroxy-cholesten-3-one	0.46	0.0341*
Histidine	Testosterone	0.69	0.0006***
Histamine	Dihydrotestosterone	0.57	0.0072**
Epinephrine	5β-Androsterone	−0.44	0.0455*
Choline	Testosterone	0.6	0.0037**
Arginine	Androstenedione	0.5	0.0203*
Arginine	Testosterone	0.68	0.0007***

Correlations were analyzed using Pearson’s test. Pearson’s r means Pearson correlation coefficient. * means P < 0.05, ** means P < 0.01, *** means P < 0.001.

## Discussion

Previous studies provided evidence for an important interplay between gonadal hormones and the neurotransmitter system (Bethea et al., 2002; Bertrand et al., 2005). Seasonal fluctuations of sex steroids in photoperiodic feed back on the brain to regulate the expression of serotonergic genes (Kranz et al., 2015; Perfalk et al., 2017). The objective of our experiment was to understand the crosstalk of neurotransmitters and steroids in seasonal reproduction under different photoperiod.

We observed that quail’s testicular morphology and functions were dramatically changed by altering the photoperiod. Photoperiod affects gonadal size and testicular activity; SD led to testis regression and reproductive failure, while LD induced gonadal mass, an increase in size, and recovery of reproductive activity. Our results confirmed previous observations that male Japanese quail transferred to different photoperiods undergo a rapid change in plasma testosterone and testis weight (Oishi and Konishi, 1978; Wada, 1993; Henare et al., 2011). The increase in gonadal mass is functional in terms of sperm and reproductive hormone production (Bauchinger et al., 2007).

As previously reported, exposure of male quail to a short photostimulation for 5 d results in a significant regression of the reproductive system (Oishi and Konishi, 1978). We observed that testosterone was significantly reduced when day length was artificially shortened; low levels of testosterone and androstenedione were observed compared to LD conditions. Meanwhile, plasma levels of testosterone could not be detected when the quail transferred to SD conditions for 14 d. In male quail, transfer from SD to LD causes significant changes in plasma LH, FSH, testosterone concentration, and testis weight (Henare et al., 2011). However, transfer from SD to LD caused significant increases in androstenedione; plasma levels of both testosterone and androstenedione were increased after 3 d. Our data showed that testicular function recovered on the third day when quail were moved from SD to LD. Steroid hormones are the principal index of reproduction. Androstenedione is a precursor of testosterone, which also has weak androgenic activity in the reproductive system (King et al., 1999). Both androstenedione and testosterone are also potential biomarkers of pregnancy in humpback whales (Luche et al., 2020).

Prior work demonstrated that a changing photoperiod can alter neurotransmitter content in the hypothalamus of the adult rat brain (Porcu et al., 2018, 2020). Chronic exposure to different photoperiods alters the number of neurotransmitters in the hypothalamus and results in behavioral changes (Pritchard et al., 2020). In the current study, the effects of photoperiod on hypothalamus neurotransmitter concentration were investigated and results indicated that the photoperiod influenced neurotransmitter content. Both arginine vasopressin (AVP) and GABA were significantly increased when day length was artificially shortened. AVP is involved to circadian time keeping, learning, and memory in the brain (Li et al., 2017; Maejima et al., 2021). GABA is an inhibitory neurotransmitter, which has long been implicated as one of the major players in modulating GnRH neurons and LH secretion (Watanabe et al., 2014; Silva et al., 2019), the content of GABA in SD_28d was significantly increased over that in LD_28d. Serotonin is related to light exposure and is widely involved in the regulation of GnRH activity (Bhattarai et al., 2014). In our results, serotonin and epinephrine were significantly reduced when day length was artificially shortened. Serotonin has been shown to be involved in reproductive function such as sexual behavior and GnRH release (Prasad et al., 2015; Han et al., 2017). Moreover, photoperiod was able to modulate epinephrine contents, and hypothalamic epinephrine concentrations were markedly decreased following stress (Roth et al., 1982; Mahapatra et al., 1988).

In quail, on the short days condition, the number of serotonin-ir cells differed significantly between light and dark phases, but did not differ between light and dark phases on long days condition (Haida et al., 2004). Long photoperiods increase serotonin neuron excitability and firing, as well as serotonin concentration in the midbrain (Green et al., 2015). When day length was artificially shortened, the concentration of serotonin was progressively reducing, but showed no change in the content of both tryptophan and 5-HIAA. However, when transferred from SD to LD, quail displayed higher levels of tryptophan but showed no change in content of both serotonin and 5-HIAA. These results suggest that mechanisms controlling hypothalamic serotonin metabolism have different regulatory modes underlying different photoperiods.

In serotonergic neurons, tryptophan serves as the precursor for 5-HT (Höglund et al., 2019), and 5-hydroxyindoleacetic acid (5-HIAA) is a major metabolite of 5-HT in the central nervous system. Tryptophan hydroxylase (TPH2) is the initial and rate-limiting enzyme of serotonin biosynthesis, which can modulate the concentration of 5-HT *in vivo* (Sloley and Juorio, 1995; Birdsall, 1998; Walther and Bader, 2003). MAOB is a key enzyme that helps break down serotonin (Bortolato et al., 2010), and the function of SERT is to re-take up serotonin (5-HT) released through the activity of serotonergic neurons (Murphy et al., 2004) while SLC18A2 is a monoamine neurotransmitter transporter, which transports amine neurotransmitters into synaptic vesicles (Kudryavtseva et al., 2017). Hypothalamic *TPH2*, *SERT*, and *SLC18A2* expression levels were reduced during adaptation to long light exposure. In mice, long photoperiods decreased brain *TPH2* and *SERT* expression levels and reduced *TPH2* levels compared to short photoperiods (Siemann et al., 2020). However, when quail were chronically exposed to SD, we observed a downregulation of mRNA level of *TPH2*, and an upregulation of mRNA levels of *MAOB* in the hypothalamus. MAOB is necessary for inactivating the neuromodulator serotonin, which is significantly down-regulated in birds exposed to breeding conditions (Thompson et al., 2012). *MAOB* affects serotonin levels and is responsible for 5-HT degradation. Our experiment revealed the seasonal expression pattern of *MAOB*, which may generate increased levels of serotonin turnover and functional levels underlying different photoperiods. Serotonin turnover is positively correlated with sexual competence in male rhesus macaques; males with high concentration of 5-HIAA are more sexually competent (Mehlman et al., 1997).

In addition, testis steroid biosynthesis genes were influenced by photoperiod. We found that in males maintained for 7 d on LD, both *NR5A1* and *3*β*-HSD* were significantly in the regressing testis; however, when males were maintained for 7 d on SD, *NR5A1* and *3*β*-HSD* were significantly reduced in the active testis. NR5A1 was suggested as a master regulating transcription factor that plays a crucial role in gonadal development, which regulates the transcription of steroidogenic enzymes involved in gonadal steroid biosynthesis in the pituitary (Hoivik et al., 2010; Ferraz-de-Souza et al., 2011). In seasonal reproduction of the Iberian mole, gene expression levels of *NR5A1* were significantly reduced in the inactive testis (Dadhich et al., 2011). However, in quail, the expression of *NR5A1* was increased in the regressed testis (Otake and Park, 2016). It was suggested that NR5A1 action on testicular development participates in the AMH signaling system in quail.

The association between testosterone and serotonin were particularly strong in hypothalamus (Kranz et al., 2015). We observed a significant negative correlation between testosterone and serotonin in S-to-L group, while showed a positive correlation with L.Tryptophan. Testosterone treatment in some studies is showed a strong influence on SERT expression (Kranz et al., 2015), whereas our data are almost absent in S-to-L groups and only reduced SERT expression in L-to-S groups. Several studies concerned with the association of neurotransmitters, especially serotonin, with seasonal reproduction have been reported (Lambert et al., 2002; Banerjee and Chaturvedi, 2019). Moreover, numerous studies have found that steroidogenesis is closely associated with seasonal reproduction, but the correlations between neurotransmitter and steroidogenesis are still unclear. Serotonin may act and influence testicular function via the GnRH/GnIH system and their receptor system, which subsequently alter the expression of steroidogenesis genes (Banerjee and Chaturvedi, 2019).

In summary, our study explored the association between hypothalamic neurotransmitter metabolism and serum steroid hormone profiles for different photoperiods in quail. Long periods of light followed by short periods induced both androstenedione and testosterone release into serum and down regulated the expression level of *NR5A1* in the testis, as well as upregulating the concentration of Arg and GABA and down regulating the concentration of L-glutamine in hypothalamic tissues. In contrast, short periods of light followed by long periods inhibited both androstenedione and testosterone release in serum and up-regulated expression levels of *NR5A1* in the testis, followed by a decline in concentration of serotonin and increased expression of *MAOB*.

## Data Availability Statement

The original contributions presented in the study are included in the article/Supplementary Material, further inquiries can be directed to the corresponding authors.

## Ethics Statement

The animal study was reviewed and approved by the Animal Experiment Committee of Zhongkai University of Agriculture and Engineering (No. 2020090909).

## Author Contributions

XS, DJ, JP, HO, YT, and YH conceived the idea and designed the experiments. XS and YX wrote the manuscript. YX, JL, and YF performed the experiments and data analysis. DF, XH, SL, and YS helped to collection the samples. All authors read and approved the final manuscript.

## Conflict of Interest

The authors declare that the research was conducted in the absence of any commercial or financial relationships that could be construed as a potential conflict of interest.

## Publisher’s Note

All claims expressed in this article are solely those of the authors and do not necessarily represent those of their affiliated organizations, or those of the publisher, the editors and the reviewers. Any product that may be evaluated in this article, or claim that may be made by its manufacturer, is not guaranteed or endorsed by the publisher.
