# Supplementary material for: Photoperiodic Changes in Both Hypothalamus Neurotransmitters and Circulating Gonadal Steroids Metabolomic Profiles in Relation to Seasonal Reproduction in Male Quail
Source: Front Physiol. 2022 Mar 25;13:824228. doi: 10.3389/fphys.2022.824228 (PMC8993408; doi:10.3389/fphys.2022.824228)
Supplement: Supplementary file 5 [file Table_5.docx]

Table S3 List of Primers used for real-time quantitative PCR

| Genes | Primer sequence(5’-3’) | The length of the product (bp) |
| --- | --- | --- |
| *TPH2* | F:CCCATTCCCAGAGTGGAGTA | 225 |
|  | R:TCCAGCAACTGGTCTCACTG |  |
| *SERT* | F:GAAGATGTGTCGGAGGTTGC | 225 |
|  | R:GACAAAGAATTCCCTGCGCT |  |
| *MAOB* | F: GCCGAGTGAAGCTGAAGAAA | 158 |
|  | R: GGGCAAAGGTGGGTTGAAAT |  |
| *SLC18A2* | F: TCCATCGAACAGAGCCAGAG | 179 |
|  | R: TGGGTATGCTTGCCAGTGTA |  |
|  | R: TGGTTCATGTACACGGGAGA |  |
| *NR5A1* | F: TTCGGCCTCATGTGCAAGAT | 146 |
|  | R: TGGTCGAACACCAGCAGC |  |
| *3β-HSD* | F: TCTGTGCTGAAGGCAGATGG | 597 |
|  | R:CGGGACTATGGAGGCAATCC |  |
| *CYP11A1* | F:CGGCCACGCTCTTCAAGTCAG | 157 |
|  | R:AGCACCTCCTTGTTCAGGGTCA |  |
|  | R: GCGTCGGTAGGAGGAGTTG |  |
| *STAR* | F: GTGGCGGACAACGGAGATAA | 105 |
|  | R: ATTGTACACCGCATCCAGGG |  |
| *GAPDH* | F:CCTTTCTGGCAAAGTCCAAG | 201 |
|  | R:GCATCTCCCCACTTGATGTT |  |
